# A retinoic acid receptor β agonist (CD2019) overcomes inhibition of axonal outgrowth via phosphoinositide 3-kinase signalling in the injured adult spinal cord

**DOI:** 10.1016/j.nbd.2009.09.018

**Published:** 2010-01

**Authors:** Marta Agudo, Ping Yip, Meirion Davies, Elizabeth Bradbury, Patrick Doherty, Stephen McMahon, Malcolm Maden, Jonathan P.T. Corcoran

**Affiliations:** a The Wolfson Centre for Age-Related Diseases, King's College London, Guy's Campus, London SE1 1UL, UK; b MRC Centre for Developmental Neurobiology, King's College London, Guy's Campus, London SE1 1UL, UK

## Abstract

After spinal cord injury in the adult mammal, axons do not normally regrow and this commonly leads to paralysis. Retinoic acid (RA) can stimulate neurite outgrowth in vitro of both the embryonic central and peripheral nervous system, via activation of the retinoic acid receptor (RAR) β2. We show here that regions of the adult CNS, including the cerebellum and cerebral cortex, express RARβ2. We show that when cerebellar neurons are grown in the presence of myelin-associated glycoprotein (MAG) which inhibits neurite outgrowth, RARβ can be activated in a dose dependent manner by a RARβ agonist (CD2019) and neurite outgrowth can occur via phosphoinositide 3-kinase (PI3K) signalling. In a model of spinal cord injury CD2019 also acts through PI3K signalling to induce axonal outgrowth of descending corticospinal fibres and promote functional recovery. Our data suggest that RARβ agonists may be of therapeutic potential for human spinal cord injuries.

## Introduction

There are at least three causes for the lack of axonal outgrowth of CNS neurons after spinal cord injury. First, the presence of growth inhibiting molecules, including Nogo-A, myelin-associated glycoprotein (MAG) and oligodendrocyte myelin glycoprotein (Omgp) (He and Koprivica, 2004). Second, insufficiency of growth-promoting factors, which are well known for their ability to promote neurite outgrowth in vitro and induce some axonal outgrowth when administered to injured cord (Lu et al., 2004; Schnell et al., 1994). Third, the lack of an appropriate ‘growth programme’ by damaged neurons (Kwon and Tetzlaff, 2001). One factor that can induce such a growth programme is retinoic acid (RA) signalling. This is mediated by retinoic acid receptors (RARs) and retinoid X receptors (RXRs), both of which have three subtypes (α, β and γ) and various isoforms (Bastien and Rochette-Egly, 2004). Transcription occurs when RA binds to an RAR/RXR heterodimer, which then binds to retinoic acid response elements (RAREs) located in the regulatory regions of target genes (Bastien and Rochette-Egly, 2004).

It has previously been shown that RARβ2 is required for retinoid mediated neurite outgrowth. Activation of RARβ2 by retinoids results in neurite outgrowth of cultured embryonic dorsal root ganglia (DRG), spinal cord and adult DRG (Corcoran et al., 2000; Corcoran et al., 2002; Corcoran and Maden, 1999; So et al., 2006). In adult RARβ null mice peripheral axonal regeneration is impeded (So et al., 2006) and when RARβ2 is transduced into cultured adult rodent spinal cord explants, which do not express this receptor, neurite outgrowth occurs (Corcoran et al., 2002). The axons from corticospinal tract (CST) neurons form the major descending pathway in the dorsal columns of the spinal cord and their damage results in functional impairments of some motor tasks. Recently it has been demonstrated that overexpression of RARβ2 by lentiviral vectors in adult DRG or CST neurons results in outgrowth of axons and functional recovery in models of CNS injury (Wong et al., 2006; Yip et al., 2006).

A much simpler approach to upregulate RARβ2 expression in vivo is to use an RARβ agonist as the gene for this receptor contains an RARE, resulting in autoregulation (Leid et al., 1992). Also, it is a much more practical solution to treat CNS injuries with a RARβ agonist than with gene therapy, since it is a small lipophilic molecule which can potentially reach all the injured neurons, and the dose can be controlled. We show here that a RARβ agonist (CD2019) leads to axonal outgrowth and functional recovery in a rodent model of spinal cord injury.

## Material and methods

### Animal surgery

All animal experiments were carried out under UK home office regulations. Dorsal column lesions (DCL) were performed on adult male rats as previously described (Bradbury et al., 2002). Miniosmotic pumps flow rate of 0.5 μl/h for 14 days (Alzet) were filled with 10 μM RARβ agonist (CD2019, obtained from CIRD Galderma, Sophia-Antipolis, France), or vehicle (10% DMSO in PBS). This gives a dose of CD2019 of 180 ng/kg/day. The pumps were placed subcutaneously and connected to a brain infusion catheter (Alzet), which was inserted into the lateral ventricle (Bregma coordinates: rostrocaudal: -0.8 mm, mediolateral: -1.5 mm and dorsoventral: -4.5 mm). CD2019 is 5-fold selective for RARβ over RARα and 12-fold selective for RARβ over RARγ (Bernard et al., 1992; Delescluse et al., 1991). The dose was based on our previous in vivo studies on activation of RARα and β signalling in the adult rat brain (Goncalves et al., 2009). Animals which underwent behavioural studies and subsequent tracing (*n* = 6 per treatment) were kept for 6 weeks after lesion before being sacrificed with a lethal injection of pentobarbital and transcardially perfused with 4% paraformaldehyde (PFA). Dissected tissue (cervical and lumbar spinal cord) was processed for immunofluoresence.

### Western blotting

Protein was extracted from the cortex of adult rats 14 days post-surgery (*n* = 3 per group). The amount of protein was determined using a bicinchoninic acid (BCA) protein assay kit (Pierce). Protein (10 μg) was loaded on 10% or 6% SDS-PAGE gel. Semidry blotting was performed, and the blots were probed with rabbit anti-RARβ (Santa Cruz, dilution of 1:500), rabbit anti-phospho-Akt, rabbit anti-Akt (both from Cell Signalling Technology, dilution of 1:1000), and mouse anti-GFAP (Sigma, dilution 1:1000). The membranes were then incubated with HRP-conjugated secondary antibodies (anti-mouse IgM+A 1:5000 from Abcam and anti-mouse and anti-rabbit from Amersham Pharmacia Biotech 1:5000) and HRP activity was visualized by applying chemiluminescent substrate (ECL; Amersham Pharmacia Biotech) followed by exposure of the membrane to X-ray film. For a loading control, the blots were probed with mouse anti-βIII tubulin (Promega, dilution of 1:1000) and developed as above. The exposed films were analyzed by Gene Tools program (Syngene). Signal density was calculated as the ratio of signal intensity to β-III tubulin.

### RT-PCR

RNA was isolated and cDNA synthesis was carried out as previously described (Corcoran et al., 2000). For PCR of rat RARβ2 (Accession no. AJ002942) the following primers were used, forward, ttcgtggacttttctgtgc and reverse, tgtagaaatccaggatctgcc, which yields a product of 134 bp. These primers are rat RARβ2 specific and cannot therefore detect other RAR/RXR isoforms. Thirty cycles were carried out using the following conditions, 94 °C for 30 s, 56 °C for 30 s and 72 °C for 30 s.

### Neurite outgrowth assays

Cerebellar neurons isolated from post-natal day 3 rat pups were cultured over monolayers of parental 3T3 cells in control media or media supplemented with a recombinant MAG-Fc chimera (R&D Systems) used at a final concentration of 20 mg/ml MAG-Fc. The monolayers were established for 24 h prior to addition of the neurons and the cocultures were maintained for approximately 21 h. CD2019 the PKA inhibitor, KT5720 (Sigma) and the PI3K inhibitor, LY294002 (Sigma) were made up at 1000× concentrations in DMSO and were added at the time of neuronal plating. Following fixation with 4% PFA, the neurons were immuno-stained with a GAP-43 antibody (a gift from Graham Wilkin, Imperial College, dilution of 1:500) and the mean length of the longest neurite per cell was measured for approximately 120–150 neurons as previously described (Williams et al., 2005).

DRG and cortical explants were obtained from adult rats, they were cultured in cellogen as previously described (Corcoran and Maden, 1999). Three explants per treatment were used. Neurite outgrowth was assessed 3 days later by immunohistochemistry with NF200 (Sigma, dilution of 1:200). The average lengths of the neurites were measured using image-pro plus software. Media consisted of DMEM-F12 (Invitrogen) containing N2 (Invitrogen) supplemented with glucose (33 mM) and glutamine (2 mM).

### Labelling of CST neurons/tract and immunohistochemistry

Descendent CST axons were anterogradely traced after DCL by injecting 10% biotin dextran amine (BDA, Mw10K from Molecular Probes) in PBS into the motor cortex as previously described (Yip et al., 2006). Six injections were done in the right cortex, (0.5 μl of BDA/injection point). Animals (*n* = 6 per treatment) were perfused and the spinal cord was transferred to PBS (plus 0.1% sodium azide) and embedded in gelatin (10%, 300 bloom; Sigma, Poole, UK). Gelatin blocks were hardened in 4% PFA, and 40 μm free-floating serial transverse sections were cut on a vibratome (Leica, Nussloch, Germany) and collected in 24-well plates containing PBS (plus 0.1% sodium azide).

BDA was detected using the tyramide amplification kit (Perkin-Elmer) coupled with extra-avidin-FITC (Amersham Pharmacia Biotech, UK, 1:500). All BDA-labelled fibres observed within a 1-mm square grid were counted at measured intervals from 5 mm above to 5 mm below the lesion site by an experimenter, blinded to treatment. BDA positive axons were counted in every third section (5 sections per animal at each point analysed, and a total of 40 sections per animal) at the same medio-lateral distance from the midpoint (as seen by the central canal).

CST neurons were labelled by retrograde tracing using Fluorogold (FG, Molecular Probes). Two microliters of 5% of FG was injected 2 mm deep into the cervical spinal cord (C3–C4) at a rate of 0.5 μl/min (*n* = 3 rats per group). In sham animals FG was injected 0.5 mm bilateral to the medial line of spinal cord (1 μl per side), and in lesioned animals FG (2 μl) was injected into the injury. After 14 days the cortices were fixed for 2 h in 4% PFA, embedded in OCT compound and stored frozen. Twelve-micrometer sagittal sections were cut and four sequential slides containing two sections from lateral 3.4 to 3.9 mm (Paxinos and Watson, 2002) were taken for analysis.

Immunohistochemistry was carried out using anti-rabbit phospho-Akt (Cell Signalling technology, dilution of 1:100). Secondary antibody used was anti-rabbit Cy3 conjugated (Jackson, used at 1:1000). Images were captured at 100× magnification using a Roperscientific digital camera.

### Measuring lesion volume

The same sections used for CST quantification were used, and every third section analysed by an observer blinded to the experimental treatment. They were studied under darkfield microscopy, and the border of the damaged area was determined. The outlined area of each animal were subsequently imported into Microsoft Excel (Seattle, WA), summed, multiplied by the section thickness, and corrected for the total number of sections.

### Behavioural testing

The behavioural tests were carried out as previously described (Bradbury et al., 2002). Rats (*n* = 6 per treatment group) were first trained for 2 weeks before surgery to perform grid walk and beam walk; they were then tested by an observer blinded to the experimental treatment once a week for 5 weeks after lesion.

### Graphs and statistics

Graphs were plotted using Sigma plot. Data are expressed as mean ± S.E.M., and statistical analysis was carried out using Student's *t*-test using Sigma Stat software (SPSS Software Ltd., Birmingham, UK). Means, SEM, SD and *P* values are provided as summary statistics.

## Results

### CD2019 increases RARβ expression in the cortex of CST lesioned rats

**Fig. 1:**
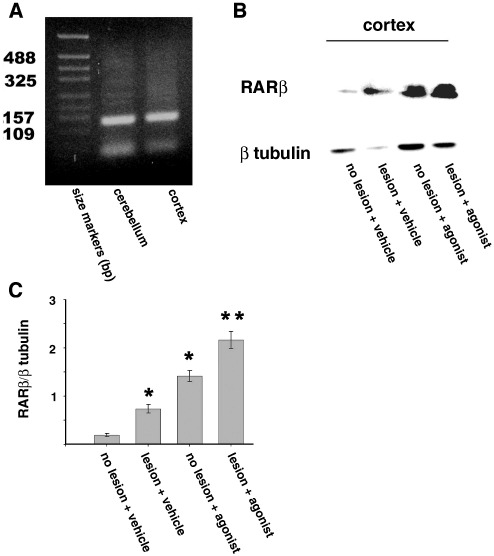
RARβ2 expression is induced by CD2019 in the adult brain. Rats were treated at the time of lesion by i.c.v. with 180 ng/kg CD2019/day for 14 days. (A) RT-PCR analysis of RARβ2 in normal adult rat cerebellum and cerebral cortex. (B) By Western blotting, there is an increase in the amount of RARβ protein detected in the cortex of CD2019-treated rats compared to the other groups. (C) Quantification of RARβ protein in the cortex. Error bars show SEM, asterisks denote significant difference between non-lesioned vehicle-treated animals and other treatments. ⁎*P* < 0.005, Student's *t*-test, *n* = 3 from three different animals for each treatment group.

We first asked if RARβ2, which is known to be involved in axonal outgrowth (Corcoran et al., 2002; So et al., 2006;Wong et al., 2006), was present in the adult brain. By RT-PCR, RARβ2 could be detected in the adult cerebellum and cortex (Fig. 1A). Since a retinoid is required to activate the RARβ2/RXR heterodimer, it is possible that the lack of axonal outgrowth in an CNS injury may in part be associated with insufficient levels of the appropriate ligand, as previous work has shown that the retinoid profile of the brain varies during development and that retinoids show a varied distribution in the adult brain (Goncalves et al., 2009;Horton and Maden, 1995). In order to investigate this we asked if the administration of CD2019 in vivo could upregulate the receptor in the injured CST neurons. Adult rats received a cervical (C4) DCL and at the time of injury 180 ng/kg/day of CD2019 was administered into the lateral ventricle by an osmotic minipump for 14 days. In the non-lesioned vehicle-treated cerebral cortices some RARβ protein could be detected, which increased 3-fold in response to the lesion. However, following treatment with the RARβ agonist there was a 4-fold increase in the non-lesion and 6-fold increase in the lesion treated compared to the control animals (Figs. 1B and C).

**Fig. 2:**
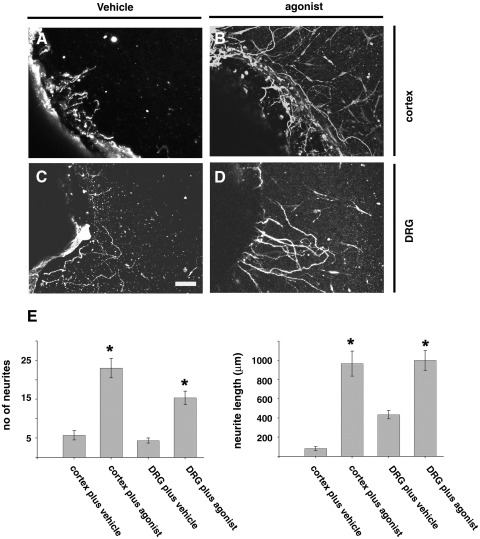
Neurite outgrowth is stimulated from cortex and DRG explants obtained from lesioned animals treated with CD2019. Animals were treated at the time of lesion by i.c.v. with 180 ng/kg CD2019/day for 14 days. Neurite outgrowth from explants of (A) cortex from lesion animals plus vehicle, (B) cortex from lesion animals treated with CD2019, (C) DRG from the region of the lesion treated with vehicle, (D) DRG from the region of the lesion treated with CD2019, (E) quantification of neurite outgrowth. There are significantly more neuritis, and their lengths are longer from explants of both cortex and DRG obtained from lesioned animals treated with CD2019 compared to vehicle-treated ones. Error bars show SEM, asterisks denote significant difference between vehicle and CD2019 treatment. ⁎*P* < 0.005, Student's *t*-test, *n* = 3 explants from three different animals. Scale bar: 100 μm.

Fourteen days after surgery and CD2019 treatment, explants of adult cortex and DRG from the lesion site were obtained from these animals and cultured in a cellogen matrix for 3 days in serum-free medium and analysed for neurite outgrowth with NF200. While there was little or no neurite outgrowth from DRG and cortex from lesioned vehicle-treated animals (Figs. 2A, C and E) there was significant increase in both neurite number and length from DRG and cortex from rats with DCL treated with CD2019 (Figs. 2B, D and E). This shows that the agonist administered in vivo is freely available to the injured neurons to induce neurite outgrowth and that in the case of the cortex an inhibitory environment can be overcome by RARβ signalling.

### CD2019 induces neurite outgrowth in vitro via PI3K signalling

**Fig. 3:**
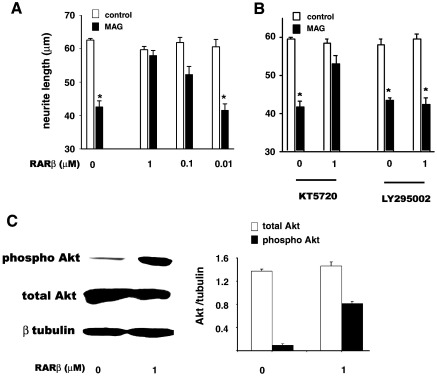
CD2019 stimulates neurite outgrowth of cerebellar neurons in the presence of MAG via PI3K signalling. (A) CD2019 overcomes MAG inhibition. Cerebellar neurons were cultured over monolayers of 3T3 cells in control media (open bars) or media supplemented with the MAG-Fc at 20 μg/ml (black bars) for 21 h. In the absence of CD2019, MAG inhibits neurite outgrowth, but with increasing concentration of CD2019 this inhibition is overcome. (B) CD2019 induced loss of inhibition is counteracted by the presence of a PI3K inhibitor (LY294002 at 10 μM) but not a PKA inhibitor (KT5720 at 200 nM). (C) Western blot for phospho-Akt and total Akt and quantification. In CD2019-treated cultures there is 4-fold induction of phospho-Akt compared to control cultures but no significant increase in total Akt. Data is representative of three different cultures. Error bar shows SEM. ⁎*P* < 0.005, Student's *t*-test.

We next asked if RARβ signalling could overcome MAG inhibition of neurite outgrowth. In the presence of MAG-Fc (20 μg/ml) the amount of neurite outgrowth from cultured P3 cerebellar neurons was inhibited by approximately 32% (Fig. 3A). This inhibition was completely overcome, in a dose-dependent manner by CD2019 (Fig. 3A). Pathways that are known to stimulate neurite outgrowth include cyclic AMP (cAMP)-dependent protein kinase A (PKA) and phosphoinositide 3-kinase (PI3K), and these are able to overcome myelin inhibition (Cai et al., 2001; Williams et al., 2005). Therefore, we asked if the RARβ signalling pathway was linked to either of these pathways. Whereas the ability of CD2019 (1 μM) to circumvent myelin inhibition was only slightly attenuated by the PKA inhibitor, KT5720 (Fig. 3B), it was significantly blocked by the PI3K inhibitor, LY294002 (Fig. 3B). Western blots of cerebellar cultures treated with 1 μM CD2019 showed a significant 4-fold increase in phospho-Akt, but not total Akt, a target of PI3K, compared to control cultures (Fig. 3C). This suggests that CD2019 acts via the PI3K pathway in stimulating neurite outgrowth by increasing the phosphorylation of Akt but not the total pool of Akt.

### CD2019 induces PI3K signalling in cultured cerebellar neurons and in lesioned CST neurons

**Fig. 4:**
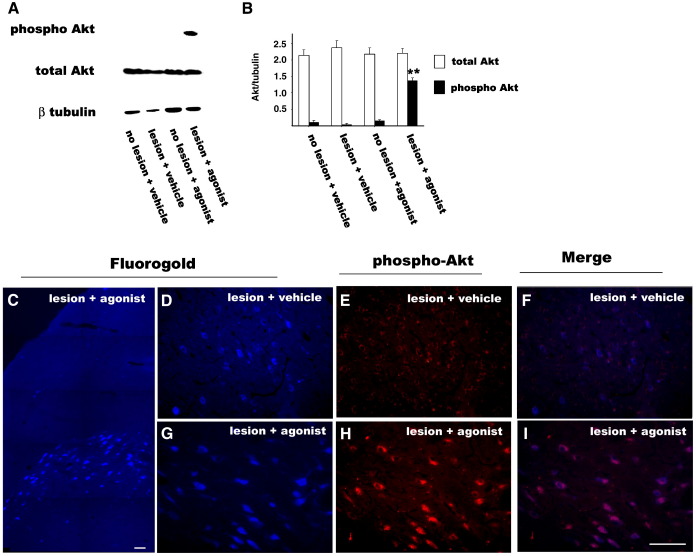
CD2019 induces phospho-Akt expression in CST neurons of lesioned rats. Rats were treated at the time of lesion by i.c.v. with 180 ng/kg of CD2019/day for 14 days. Western blotting of phospho-Akt (A) and quantification (B). In the cortex of the lesioned rats treated with CD2019 phospho-Akt is induced 8-fold compared to vehicle-treated. (C–G) FG and phosho-Akt labelling of the CST neurons, (C) low power of FG labelling of layer V of the cortex, (D–F) in vehicle-treated brains there is lack of phospho-Akt expression in the CST neurons, (G–I) in contrast in CD2019-treated brains phospho-Akt is induced in the CST neurons. Error bars show SEM, asterisks denote significant difference between vehicle and CD2019 treatment. ⁎*P* < 0.005, Student's *t*-test, *n* = 3 from three different animals. Scale bar = 50 μm.

We next asked if CD2019 could induce phospho-Akt expression in the cortex of the CST lesioned animals. The same in vivo dose of drug was used as above and administered for 14 days. By Western blotting very low amounts of phospho-Akt were detected in the non-lesioned vehicle-treated animals, and this was not increased in the vehicle-treated lesioned or non-lesioned CD2019-treated animals. However, in the lesioned CD2019-treated animals there was an 8-fold increase in the amount of phospho-Akt compared to the lesioned vehicle-treated (Figs. 4A and B).

In order to confirm that phospho-Akt was induced by CD2019 in the injured CST neurons, they were retrogradedly labelled with Fluorogold (Figs. 4C–E) at the time of injury and analysed 2 weeks later. By immunohistochemistry, phospho-Akt was not detected in the CST neurons of the vehicle-treated rats (Figs. 4D–F) but was co-localised in the CST lesioned neurons of the CD2019-treated animals (Figs. 4G–I). These data indicate that RARβ signalling is acting via PI3K/Akt signalling pathway, as we have shown in vitro and that this only occurs in the injured neurons.

### CD2019 induces axonal outgrowth from lesioned CST neurons

**Fig. 5:**
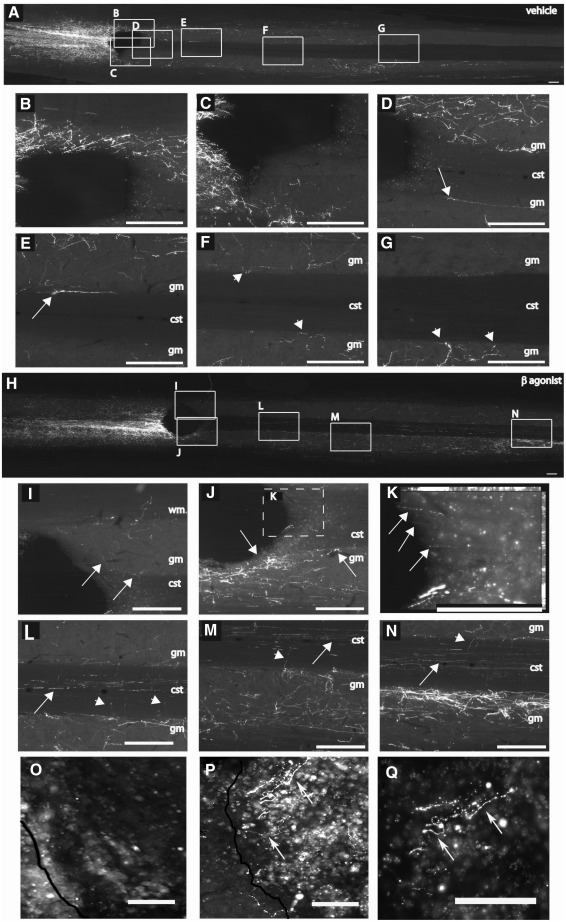
CD2019 promotes growth of CST axons following lesion. Rats were treated at the time of lesion by i.c.v. with 180 ng/kg CD2019/day for 14 days and then analysed 28 days later. Axonal outgrowth was determined with BDA tracing in longitudinal sections of spinal cord rostral (left) and caudal (right) to the lesion site. In vehicle-treated rats BDA-labelled axons were apparent along the CST, rostral to the injury (A–C), but absent caudal to it (D–G). BDA-labelled fibres can be seen in adjacent grey matter, running parallel (D, E, arrows) or diverting away (F–G, arrowheads) from the white matter region of the injured CST. In contrast, CD2019-treated rats exhibited BDA-labelled fibres in the CST caudal to the lesion site (H). Proximal to the lesion, BDA fibres (arrows) can be seen growing around the lesion site through the grey matter (I-J) and within the white matter region of the injured CST (K). Caudally to the injured CST, BDA-labelled fibres can be seen growing longitudinally within (arrows) or tangentially (arrowheads) to the white matter tract (L–N). In the grey matter of the forelimb innervation field many axons can be seen (N). Saggital sections of the lesion site (O–Q), in vehicle-treated animals axons cannot be detected in the lesion site (O), in contrast in CD2019-treated animals axonal outgrowth (arrows) is observed in the lesion site (P,Q). Black lines indicate the rostral part of the lesion for O–Q. Scale bars = 500 μm (A–N), 100 μm (O–Q).

We next repeated our in vivo experiment with the same dosing regime of CD2019 as above. At 5 weeks after surgery, rats were examined for axonal outgrowth by BDA labelling. In lesioned vehicle-treated rats (Figs. 5A–G), BDA-labelled CST axons could be detected rostral to the injury within the CST and collaterals branching out into the grey matter (Figs. 5A–G). Caudal to the injury site, no BDA-labelled fibres were found in the CST, but some were present in the grey matter, which are likely to be collaterals of the CST axons intact above the lesion (Figs. 5D–G). In contrast, in lesioned CD2019-treated rats (Figs. 5H–N), BDA-labelled CST axons were detected growing around the lesion and into the white matter adjacent to the lesion site caudally (Figs. 5I and J) and proximal to the site of injury (Fig. 5K). Beyond the lesion many BDA-labelled CST axons could be seen along the tract and axons branching off the white matter and into the grey matter (Figs. 5L–N). At the forelimb innervation field many axons were located in the grey matter and CST axons could be seen growing both longitudinally and tangentially compared to vehicle-treated animals (Fig. 5N). While no descending CST axons could be detected in the lesion site of vehicle-treated animals (Fig. 5O), they were present in the CD2019-treated animals (Figs. 5P, Q) suggesting that it facilitates axonal outgrowth inside an inhibitory environment.

### CD2019 does not affect tissue or axonal sparing but increases axonal outgrowth

**Fig. 6:**
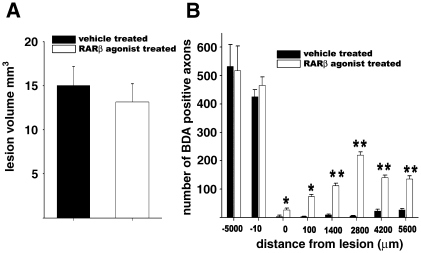
CD2019 does not affect lesion volume but promotes growth of CST axons. Rats were treated at the time of lesion by i.c.v. with 180 ng/kg CD2019/day for 14 days and then analysed 28 days later. (A) The lesion volumes are similar in both CD2019 and vehicle-treated rats, suggesting that CD2019 does not alter tissue sparing. (B) quantification of axonal outgrowth in sagittal sections of the injured cord. In the CD2019-treated animals there is a significant increase in the number of labelled BDA axons (empty bars) beyond the lesion at all distances compared to the vehicle-treated lesioned animals. Error bars show s.d. ⁎*P* < 0.05, ⁎⁎*P* < 0.005, Student's *t*-test, *n* = 6 rats for each treatment group. Asterisks denote significant difference between vehicle and CD2019 treatment.

Measurement of lesion volume 6 weeks after lesion showed there was no significant difference between the CD2019 and vehicle-treated lesioned cords (Fig. 6A), suggesting that RARβ signalling does not prevent tissue sparing, ruling out this as a reason to explain axonal outgrowth in the CD2019-treated animals. We next counted the number of BDA-labelled axons both in the white and grey matters. There were no significant differences in the number of BDA-labelled axons at 5000 μm rostral to the lesion in both vehicle and CD2019-treated animals (Fig. 6B) or at 10 μm rostral to the lesion (Fig. 6B). This suggests that RARβ signalling does not have an effect on axonal sparing. At increasing distances caudal to the lesion the number of BDA-labelled axons in the CD2019-treated animals increased significantly compared to the vehicle-treated ones (Fig. 6B). At 2800 μm beyond the lesion, which is the level of forelimb innervation, there was a significant peak in the number of BDA-labelled axons in CD2019-treated animals compared to vehicle-treated animals (Fig. 6B). These data suggest that the RARβ signalling induces axonal outgrowth.

### CD2019 induces functional forelimb recovery in CST lesioned rats

**Fig. 7:**
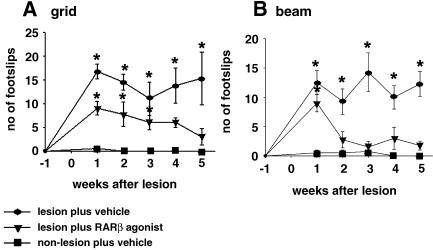
CD2019 induces functional recovery of the forelimb in lesioned animals. Rats were treated at the time of lesion by i.c.v. with 180 ng/kg CD2019/day for 14 days. (A) In a grid and (B) beam test, CD2019-treated lesioned rats showed functional recovery 4 weeks post-lesion in a grid task and 2 weeks post-lesion in a beam task, whereas there was no significant recovery in the vehicle-treated lesioned animals. Error bar shows SEM. Asterisks denote significant difference between the lesioned treated (CD2019 or vehicle) and non-lesioned vehicle-treated group ⁎*P* < 0.05, Student's *t* test, *n* = 6 rats for each treatment group.

To assess forelimb recovery, rats were tested once a week in behavioural tasks, which measured sensorimotor function over a period of 5 weeks after lesion. Non-lesioned vehicle-treated rats made virtually no footslips during the 5-week period when they walked across a narrow grid or beam (Figs. 7A and B) neither did non-lesioned rats administered with the RARβ agonist (data not shown). CST lesioned rats treated with the vehicle made significantly more footslips on the grid and beam throughout the course of the experiment (5 weeks) compared to vehicle-treated non-lesioned rats (Figs. 7A and B). In contrast, CST lesioned rats treated with CD2019 showed functional recovery on the grid 4 weeks post-lesion and 2 weeks post-lesion on the beam, as the number of foot slips were no longer significantly different to vehicle-treated non-lesioned rats at these two time points (Figs. 7A and B).

## Discussion

Our results suggest that one reason for failure of axonal outgrowth in the injured CNS is that there is loss of RA-driven signalling pathways and that this arises because of a lack of appropriate ligand to activate RARβ2/RXR heterodimers. Recently it has been shown that the distribution of retinoids varies at different stages of development and in different parts of the brain (Goncalves et al., 2009; Horton and Maden, 1995). Genes associated with axonal regeneration may be upregulated in response to CST lesions, as some sprouting of the neurons occurs without any further intervention (Caroni and Schwab, 1988; Schnell and Schwab, 1990). We have shown here that RARβ2 is induced in response to injury, but it is only when the ligand is present that axonal outgrowth and functional recovery of the forelimb occurs.

CD2019 can cause an increase in the expression of RARβ2 in the neurons themselves in the lesioned animals, as the promoter of the RARβ gene contains a RARE which can bind RARβ2 and thus autoregulate itself (Leid et al., 1992). CD2019 can be administered at the time of spinal cord injury, and as it is a small lipophilic molecule, it can reach all the injured neurons which express RARβ2. This includes the DRG (Corcoran et al., 2000; Corcoran and Maden, 1999; So et al., 2006), and we have shown here that explants of DRG from CD2019-treated lesioned animals have enhanced neurite outgrowth. Retinoids including RARβ agonists have also been shown to stimulate proliferation and differentiation of neural progenitor cells (Goncalves et al., 2005, 2009; Haskell and LaMantia, 2005; Wang et al., 2005). Thus other RARβ responsive neural cells may contribute to the functional recovery we see in the CD2019-treated lesioned animals.

In addition some of the behavioural recovery we see in the CD2019-treated lesioned rats may occur from increased sprouting from spared axons. The model we have used here the DCL is known to leave the ventral CST intact. This makes up about 5% of the total CST and undergoes collateral sprouting leading to spontaneous functional recovery in models of spinal cord injury (Weidner et al., 2001). As 65% of human spinal cord injuries are incomplete lesions, any factor that can increase axonal sprouting may be beneficial (Raineteau and Schwab, 2001).

The administration of CD2019 is a simpler approach than using lentiviral vectors overexpressing RARβ2 (Wong et al., 2006; Yip et al., 2006) for axonal outgrowth and overcomes three major disadvantages. Firstly, the vector may not transduce all injured neurons, secondly, it may insert randomly into the genome causing deleterious effects, and thirdly, it cannot be switched off once it has carried out it's role in neuronal repair. It remains to be shown at what other time points after spinal cord injury if CD2019 can give axonal outgrowth and the optimal dosing period.

CD2019 can promote neurite outgrowth within the MAG inhibitory environment in vitro. While we have not directly shown what axonal inhibitory pathways are overcome by CD2019 in vivo, it does act as in vitro via PI3K signalling suggesting that this pathway is essential for axonal outgrowth. The PI3K signalling pathway has been shown to be involved in neurotrophin-mediated axonal elongation and guidance (Atwal et al., 2000; Ming et al., 1999). One of the downstream targets of this pathway is phospho-Akt, and we have shown here that it increases in response to RARβ signalling both in cultured neurons and the lesioned neurons in vivo, suggesting that the same pathway operates both in vivo and in vitro. A similar activation of the PI3K pathway by retinoids has also been shown in neuroblastoma and MCF-7 cells and this requires the presence of a cytoplasmic located RAR which interacts directly with PI3K (Lopez-Carballo et al., 2002; Ohashi et al., 2009).

We have shown here that phospho-Akt accumulates in the neuronal cell bodies of the CST which are located in the cortex, these are the neurons which are injured in our model of spinal cord injury. It is interesting to note that phospho-Akt is only upregulated by CD2019 in the injured neurons. Previous data has also shown the importance of Akt signalling in neurite outgrowth. In vivo loss of Akt signalling leads to defects in neurite outgrowth from the cortex and this is due to defects of laminin γ1 expression (Chen et al., 2009). The induction of phospho-Akt also occurs in neuronal cell bodies and the axonal growth cones in response to NGF (Zhou et al., 2004), and it has previously been shown that NGF can stimulate RARβ signalling (Corcoran and Maden, 1999).

One therapeutic option for spinal cord injury is to prevent the wave of secondary cell death which increases the size of the lesion (Giulian and Robertson, 1990; Means and Anderson, 1983; Tator and Fehlings, 1991). Such tissue sparing occurs when Schwann cells or olfactory ensheathing cells are grafted into the lesion (Ruitenberg et al., 2003; Takami et al., 2002). However, CD2019 does not increase tissue sparing as the lesion volumes are not significantly different between agonist and vehicle-treated animals. Nor does CD2019 increase axonal sparing which can lead to functional recovery (Pearse et al., 2004), as there is no significant difference with the vehicle-treated animals in the number of axons at either 5000 or 10 μm rostral to the lesion.

Various treatments for spinal cord injury have been proposed, including growth factors (Murray et al., 2004), anti-inflammatory molecules (Eng and Lee, 2003), stem cells (Myckatyn et al., 2004) and gene therapy (Romano, 2003). We propose that retinoids are another potential treatment, and they have the distinct advantage in that they are easily administered to the injured neurons, as they are small lipophilic molecules which can cross the blood brain barrier.
